# Antimicrobial Central Venous Catheters vs. Uncoated Central Venous Catheters in Reducing Catheter-Related Bloodstream Infections in ICU: A Retrospective, Multicenter Study

**DOI:** 10.3390/medicina62061105

**Published:** 2026-06-06

**Authors:** Vincenzo Pota, Francesco Imperatore, Rossella Esposito, Chiara Cafora, Ludovica Golino, Giovanni Liguori, Fiora Silvestro, Maria Beatrice Passavanti, Pasquale Sansone, Maria Caterina Pace, Francesco Coppolino

**Affiliations:** 1 Department of Women, Child, General and Specialistic Surgery, University of Campania “L. Vanvitelli”, 80138 Napoli, Italy; fiora.silvestro@gmail.com (F.S.); mariabeatrice.passavanti@unicampania.it (M.B.P.); pasquale.sansone@unicampania.it (P.S.); mariacaterina.pace@unicampania.it (M.C.P.); francesco.coppolino@unicampania.it (F.C.); 2 Intensive Care Unit, AORN “Cardarelli”, 80131 Napoli, Italy; francesco.imperatore@aocardarelli.it (F.I.); rossella.esposito@aocardarelli.it (R.E.); chiara.cafora@aocardarelli.it (C.C.); giovanni.liguori@aocardarelli.it (G.L.); 3 Anaesthesia and Post-Operative Intensive Care Unit, AORN dei Colli—Monaldi Hospital, 80131 Napoli, Italy; ludovica.golino00@gmail.com

**Keywords:** catheter-related bloodstream infection, central venous catheter, chlorhexidine–silver sulfadiazine

## Abstract

*Background and Objectives*: Central venous catheters (CVCs) are widely used in intensive care units (ICUs) but are associated with catheter-related bloodstream infections (CRBSIs), which increase morbidity, mortality, and healthcare costs. Antimicrobial-impregnated catheters, including chlorhexidine–silver sulfadiazine (CSS)-coated CVCs, have been proposed to reduce this risk. This study evaluated the effectiveness of CSS-coated CVCs in preventing CRBSIs in ICU patients. *Materials and Methods*: A retrospective multicenter study was conducted in two ICUs in Naples, Italy. Patients admitted between October and December 2020 who received standard uncoated CVCs (Group A) were compared with patients admitted between October and December 2021 who received CSS-coated CVCs (Group B). Inclusion criteria were age 18–89 years, ICU admission with CVC placement, and negative blood cultures at admission. The primary outcome was the incidence of CRBSI, defined according to microbiological criteria consistent with current guidelines. The secondary outcome was the number of catheter removals due to confirmed CRBSI. *Results*: A total of 320 patients were included (170 in Group A and 150 in Group B). Baseline demographic characteristics and ICU admission diagnoses were comparable between groups. Microbiologically confirmed CRBSI incidence was significantly lower in Group B than in Group A (6.4% vs. 31.7%, *p* < 0.0001), corresponding to infection rates of 1.48 vs. 6.95 per 1000 catheter-days, respectively (*p* < 0.0001). Patients in Group B also required fewer catheter removals due to CRBSI (mean 1.6 vs. 3.2 per patient, *p* < 0.0001). Logistic regression confirmed a significantly lower risk of CRBSI with CSS-coated CVCs (OR 0.15; 95% CI 0.06–0.32). *Conclusions*: CSS-coated CVCs were associated with a significant reduction in CRBSI incidence and catheter replacement rates in ICU patients. However, given the retrospective design, univariable analysis, and highly unequal pandemic-related systemic stressors between the two periods, these findings demonstrate a clinical association rather than direct causation, and should be interpreted with caution due to potential residual confounding.

## 1. Introduction

Central venous catheters (CVCs) are commonly used in hospital settings, particularly in intensive care units (ICUs) [1]. Catheter-related bloodstream infection (CRBSI) is a major complication associated with CVC use and is linked to increased mortality, morbidity, and healthcare costs [2]. In the United States, approximately 80,000 CRBSIs occur each year in intensive care units, with an estimated rate of 5.3 per 1000 catheter-days, an attributable mortality of up to 35%, and a cost ranging from $34,508 to $56,000 per episode of infection. Across the entire hospital setting, this corresponds to as many as 250,000 cases annually, with an attributable mortality ranging from 12% to 25% for each infection [3,4].

The established literature indicates that the microorganisms most frequently isolated in bloodstream infections, including microbiologically confirmed CRBSIs, are coagulase-negative staphylococci, followed by *Enterococcus* spp., *Klebsiella* spp., and *Staphylococcus aureus* [5,6]. *Staphylococcus aureus* is the second most common pathogen responsible for bacteremia. The latest Infectious Diseases Society of America (IDSA) guidelines for the management of CRBSI recommend catheter removal or exchange in cases of *S. aureus* bacteremia [7]. However, removal of a CVC and placement of a new catheter are not always straightforward in critically ill patients, particularly in the presence of coagulopathy, thrombocytopenia, neutropenia, or other conditions that may increase the risk of catheter-related complications [8].

Since the early 2000s, a progressive reduction in CRBSIs caused by *Staphylococcus aureus* has been observed, particularly in ICUs. This trend is largely attributed to the widespread implementation of evidence-based central-line care bundles focusing mainly on insertion practices [9]. The adoption of best practices for catheter insertion and maintenance—such as maximal sterile barriers, proper CVC site disinfection, and avoidance of the femoral site when possible—represents an effective strategy to prevent CRBSI [7].

Since the 1980s, catheter impregnation with antiseptic or antibiotic agents has been developed as an additional preventive strategy. Among these technologies, chlorhexidine–silver sulfadiazine (CSS) and minocycline–rifampicin (MNR) impregnated catheters are the most widely used today [10]. Although these catheter-based technologies are promising, the increasing evidence of bacterial adaptation and antimicrobial resistance has raised concerns regarding their long-term effectiveness [11]. Recent guidelines suggest the use of chlorhexidine–silver sulfadiazine or minocycline–rifampicin impregnated CVCs in patients whose catheter is expected to remain in place for more than five days [12].

Pathogenically, the major cause of CRBSIs lies in the migration of skin-surface microorganisms along the external surface of the catheter during insertion (extraluminal route), or through hub contamination during subsequent manipulations (endoluminal route). Once inside, these pathogens adhere to the biomaterial surface and embed themselves within a self-produced extracellular polymeric substance, forming a structured biofilm. This biofilm acts as a mechanical and biological shield, drastically reducing bacterial susceptibility to systemic antibiotics and host immune defenses, thereby making the prevention of initial surface colonization the most critical target in mitigating these infections.

The aim of this study was to evaluate the effect of introducing a chlorhexidine–silver sulfadiazine (CSS)-coated CVC within the framework of a historical before-and-after observational analysis. Specifically, we aimed to evaluate how the implementation of this technology performed when transitioning from a period of peak pandemic-related systemic crisis (late 2020) to a period of relative healthcare stabilization (late 2021), acknowledging that the clinical environment operated under drastically shifting levels of organizational strain.

## 2. Materials and Methods

### 2.1. Study Design and Patient Population

This retrospective, observational, multicenter study was conducted in two ICUs in Naples, Italy. Two cohorts of patients were analyzed: those admitted between October and December 2020 who received standard uncoated CVCs (Group A) and those admitted between October and December 2021 who received chlorhexidine–silver sulfadiazine–coated CVCs (Group B). During the two study periods, the participating ICUs maintained the same basic organizational structure and formal infection prevention protocols. However, it must be explicitly highlighted that the two periods were characterized by highly unequal levels of organizational strain and systemic stress. The baseline period (late 2020) coincided with the peak of the second COVID-19 wave, which was marked by severe staff shortages, extreme clinical workloads, and logistical disruptions. In contrast, the late 2021 period represented a phase of relative healthcare stabilization. Consequently, this study should be interpreted as a before-and-after analysis under varying systemic stress conditions rather than a controlled comparison of catheter technologies alone.

The infection prevention bundle implemented in both ICUs included maximal sterile barrier precautions during catheter insertion, chlorhexidine skin antisepsis, strict hand hygiene, preferential use of the subclavian site when feasible, standardized catheter maintenance protocols, and daily evaluation of catheter necessity. These practices were routinely applied during both study periods.

• Group A included all patients admitted to the participating ICUs during the last three months of 2020 who required a central venous catheter (CVC) and received a non-medicated catheter (antimicrobial-impregnated catheters were not available in these ICUs at that time).
• Group B included all patients admitted during the last three months of 2021 who required a CVC and received an antimicrobial-impregnated catheter, specifically a chlorhexidine–silver sulfadiazine (CSS)-coated catheter.

### 2.2. Targeted CVC Selection and Clinical Protocols

Consistent with international recommendations from the CDC, GAVeCeLT, and SHEA, the use of chlorhexidine–silver sulfadiazine (CSS)-coated catheters in Group B was not implemented as a universal routine practice, but was targeted toward a high-risk cohort. Inclusion criteria for the CSS group specifically targeted patients with anticipated catheter dwell times exceeding five days, those requiring total parenteral nutrition, or those requiring emergency bedside insertion—factors recognized as independent predictors for increased CRBSI risk. By applying these selective criteria, the study aligns with guideline-based indications for antimicrobial-impregnated devices in clinical settings where institutional infection rates remain above benchmarks despite bundle compliance [13,14,15].

To ensure methodological consistency and clinical comparability between the two study periods, the analysis specifically focused on matching the high-risk profile of Group B with patients in Group A who exhibited identical clinical indications for CVC placement (e.g., expected prolonged dwell time, severity of illness, and requirement for specialized vascular access). This approach was adopted to verify that the observed differences in infection rates were attributable to the catheter technology rather than inherent differences in the patients’ baseline susceptibility to infection.

Catheter securement was standardized using institutional techniques to minimize migration and local trauma. While specific individual data on the number of lumens and the ratio of emergency versus elective insertions were not systematically available in the retrospective database, the clinical teams and emergency placement protocols remained identical between the 2020 and 2021 cohorts. Therefore, the main difference between the two cohorts was the type of catheter used (uncoated versus chlorhexidine–silver sulfadiazine–coated CVC).

The study protocol was approved by the local ethics committee (EC AOU Vanvitelli 0011610/e), and the requirement for informed consent was waived due to the retrospective and observational nature of the study. The research was conducted in accordance with the Declaration of Helsinki.

### 2.3. Data Extraction and Variable Definition

Data were extracted from electronic medical records using a standardized form and included demographic characteristics, major comorbidities, ICU admission diagnoses, history of hospitalization or antibiotic therapy in the preceding three months, colonization with multidrug-resistant (MDR) bacteria, type and site of CVC insertion, clinical presentation of CRBSI, and antibiotic treatment administered.

To ensure comparability with international surveillance standards (CDC and IDSA), the incidence of CRBSI was expressed as the number of infections per 1000 catheter-days. Given the retrospective design, total catheter-days were calculated using the ICU length of stay (LOS) as a proxy for catheter dwell time. This methodology was supported by institutional clinical practice, where patients enrolled in both cohorts required continuous central venous access for the entire duration of their ICU stay due to their clinical severity. Specifically, because this cohort exclusively comprised critically ill patients suffering from severe trauma, complex cerebral hemorrhages, or severe acute respiratory failure, continuous central access was systematically mandatory to sustain prolonged mechanical ventilation, support continuous titration of vasoactive drugs, facilitate advanced hemodynamic monitoring, and administer total parenteral nutrition. The total number of catheter-days for each group (7769 for Group A and 6750 for Group B) was obtained by summing the individual LOS of all included patients.

Baseline demographic characteristics and ICU admission diagnoses were compared between the two groups to assess potential differences in patient profiles and disease severity. The distribution of admission diagnoses and demographic variables did not significantly differ between groups, suggesting comparable baseline clinical characteristics.

Due to the retrospective design of the study, detailed information regarding all patient comorbidities and colonization status was not consistently available in the medical records and therefore could not be systematically included in the analysis.

### 2.4. Inclusion and Exclusion Criteria

Inclusion Criteria

• Age 18–89 years
• Admission to the ICU
• Placement of a CVC

**Exclusion criteria**

• Age <18 or >89 years
• Septic shock at admission
• Positive blood cultures at admission
• Known allergy to CVC components

### 2.5. Diagnostic Definitions and Primary/Secondary Outcomes

The primary outcome was the incidence of **microbiologically confirmed CRBSI** in the two patient groups. Due to the retrospective nature of the study, the exact timing of catheter-related bloodstream infection onset relative to catheter insertion was not consistently documented in the medical records. Therefore, the precise date of CRBSI events could not be systematically analyzed for all patients. Blood cultures were obtained at fever peaks or during significant increases in inflammatory markers not attributable to other clinical causes. We strictly distinguished between Catheter-Associated Bloodstream Infections (CLABSI), a surveillance-based definition, and Catheter-Related Bloodstream Infections (CRBSI), which requires definitive microbiological evidence. Three blood culture sets were collected as recommended by current guidelines. CRBSI was confirmed according to IDSA criteria when the same microorganism was isolated from both the central venous catheter and a peripheral vein, utilizing a quantitative/semi-quantitative cultures. Specifically, a growth of ≥15 colony-forming units (CFU) using the semi-quantitative roll-plate method described by Maki, or ≥10^3^ CFU using quantitative techniques, was required for confirmation. To ensure diagnostic rigor, cases meeting only the CLABSI surveillance criteria without DTP or positive tip confirmation were excluded

To ensure diagnostic accuracy, generic positive blood cultures without a concomitant positive tip culture were excluded, adhering strictly to the IDSA definition of microbiologically confirmed CRBSI.

The secondary outcome was the number of catheters removed and replaced due to confirmed CRBSI.

### 2.6. Statistical Analysis

A total of 320 patients were included in the study (170 in Group A and 150 in Group B). The normality of data distribution was assessed using the Shapiro–Wilk test. Baseline characteristics were compared between groups using Student’s *t*-test or the Mann–Whitney U test for continuous variables (expressed as mean ± SD or median and IQR, as appropriate) and the Chi-square test or Fisher’s exact test for categorical variables.

The primary outcome, the incidence of CRBSI, was analyzed using the Chi-square test and expressed both as a percentage and as infection rates per 1000 catheter-days to ensure comparability with international standards. For the secondary outcome (number of catheters removed and replaced), the Mann–Whitney U test was employed due to the non-normal distribution of the data.

A univariate logistic regression model was used to estimate the odds ratio (OR) and 95% confidence intervals (CI) for the risk of developing CRBSI in Group B compared with Group A. Statistical significance was set at *p* < 0.05. All analyses were performed using standard statistical software.

## 3. Results

### 3.1. Baseline Demographic and Clinical Characteristics

**Figure 1:**
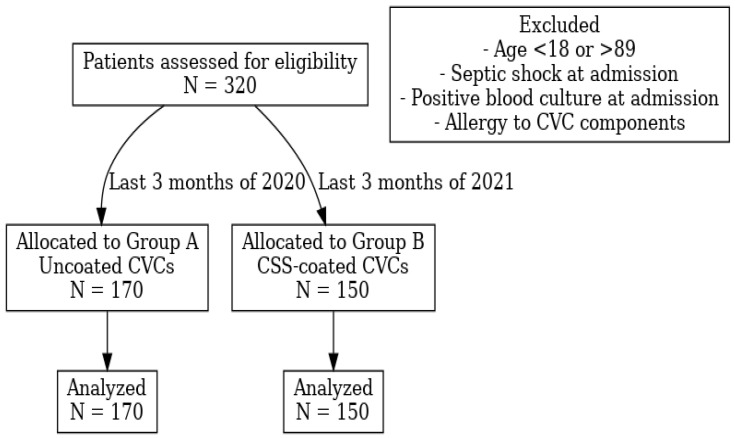
Study flow chart showing the selection process and group distribution of the 320 ICU patients included in the analysis.

A total of 320 patients were included in the study, with 170 in Group A (uncoated CVCs) and 150 in Group B (CSS-coated CVCs) (Figure 1).

Baseline demographic characteristics and ICU admission diagnoses were comparable between the two groups (Table 1), suggesting similar baseline patient profiles and disease severity.

**Figure 2:**
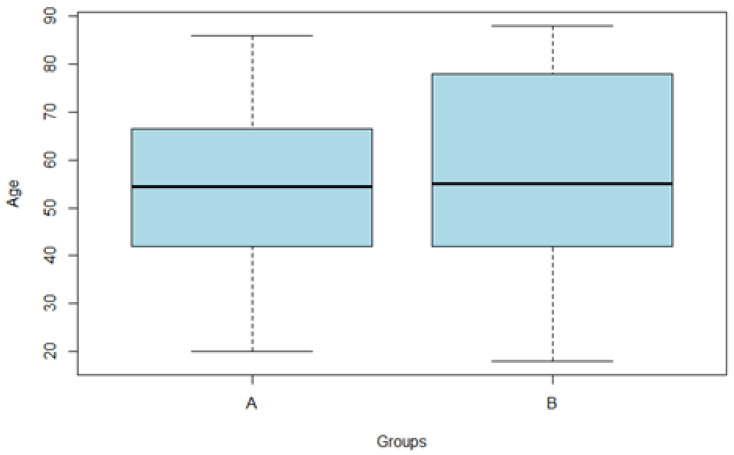
Mean Age.

**Figure 3:**
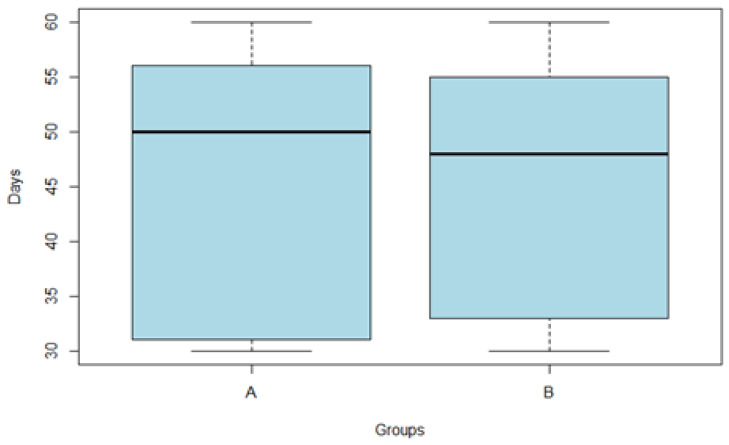
Mean lenght of ICU stay.

The mean age was 54.3 ± 18.2 years in Group A and 55.4 ± 21.3 years in Group B (*p* = 0.674) (Figure 2). The sex distribution was similar (males: 56.7% in Group A vs. 52.8% in Group B; *p* = 0.631). The mean length of ICU stay, which served as a proxy for catheter dwell time, was 45.7 ± 12.1 days in Group A and 45.0 ± 11.1 days in Group B (*p* = 0.635) (Figure 3).

### 3.2. Microbiologically Confirmed CRBSI Incidences and Management Outcomes

Clinical outcomes and infection rates are summarized in Table 2. The incidence of **microbiologically confirmed CRBSI** was significantly lower in Group B (6.4%) than in Group A (31.7%) (*p* < 0.0001). When normalized by catheter-days, the infection rate in Group B was 1.48 per 1000 catheter-days, representing a substantial reduction compared to 6.95 per 1000 catheter-days in Group A.

Patients in Group B also required significantly fewer catheter removals due to confirmed infection, with a median of 2 (IQR 1–2) vs. 3 (IQR 2–4) per patient in Group A (*p* < 0.0001). Univariate logistic regression analysis confirmed that patients receiving CSS-coated CVCs had a significantly lower risk of developing CRBSI (OR 0.15; 95% CI 0.06–0.32; *p* < 0.0001) (Table 3). his result reflects the clinical impact observed, although it should be interpreted considering the healthcare stabilization between 2020 and 2021. Because the logistic regression model was univariable and did not adjust for potential time-dependent confounders or patient-level clinical procedures, the true magnitude of the isolated protective effect remains difficult to isolate, and the reported OR may overstate the catheter’s independent efficacy due to residual confounding.

ICU admission diagnoses were also comparable between the two groups (*p* = 0.4373).

Clinical outcomes are summarized in Table 2.

### 3.3. Pathogen and Microbiological Distribution

Regarding microbiological data, the distribution of pathogens was similar across both periods. Gram-positive organisms accounted for the majority of isolates, with *Staphylococcus aureus* being the most frequent (78% in Group A and 81% in Group B). Other isolates included Gram-negative bacteria and multidrug-resistant (MDR) organisms.

## 4. Discussion

Invasive catheter infections are a leading cause of hospital-acquired infections [2]. Catheter-related bloodstream infections (CRBSIs) occur mainly through two mechanisms. The first involves the insertion site, where skin pathogens contaminate the external surface of the catheter tract and migrate to the catheter tip, reaching the bloodstream (extraluminal route). The second route of contamination is endoluminal and occurs when the catheter hub is manipulated. Pathogens colonize the internal surface of the catheter, adhere to it, and become incorporated into biofilm. A less common route is hematogenous spread from a secondary bloodstream infection. Both extraluminal and endoluminal colonization depend on the interaction between the biological characteristics of the pathogen and the surface properties of the catheter [16].

This explains why impregnated catheters may play an important role in reducing CRBSIs. Importantly, the two study periods were conducted in the same ICU settings, with the same clinical teams and infection prevention protocols. Therefore, differences in infection rates are unlikely to be explained by variations in staff training or adherence to catheter care bundles. In our experience, the introduction of CSS-coated antimicrobial CVCs was associated with a marked reduction in the percentage of **microbiologically confirmed CRBSI** and in the absolute number of catheters removed due to this infection. The implementation of strict aseptic techniques, together with the use of CSS-coated antimicrobial catheters, represents a cornerstone in the prevention of bloodstream infections in critically ill patients.

The efficacy of CSS-coated catheters is further supported by the latest evidence-based guidelines, which emphasize that antimicrobial-impregnated devices are strongly indicated when institutional infection rates remain above benchmarks despite bundle compliance [17]. These updated standards reiterate the importance of a multifaceted approach: combining site selection (prioritizing the subclavian vein), maximum sterile barrier precautions, and routine ultrasound guidance to minimize insertion trauma and subsequent colonization. Our study confirms that integrating CSS technology into these established protocols provides a significant protective advantage in the ICU environment [17]. The protective effect of CSS-coated catheters is particularly relevant in the ICU population, where multiple independent risk factors for CRBSI coexist, including the frequent use of total parenteral nutrition, renal replacement therapy, and the need for multi-lumen access. Although our study could not provide a patient-by-patient adjustment for each of these clinical variables, the consistency of the clinical protocols between the two study periods suggests that the observed reduction in infection rates is primarily attributable to the antimicrobial technology rather than shifts in patient management or catheter characteristics.

We must acknowledge that the two study periods coincided with different phases of the COVID-19 pandemic. The control group (Group A) was enrolled during the peak of the second wave in late 2020, a period associated with significant staff overload and potential challenges in maintaining strict bundle compliance. This likely explains the high baseline infection rate observed (6.95 per 1000 catheter-days). While the temporal bias is a significant factor, the fact that baseline patient characteristics and admission diagnoses remained comparable between the two cohorts suggests that the use of CSS-coated catheters provided an essential additional layer of protection.

A critical consideration in interpreting our findings is the significant temporal bias introduced by the COVID-19 pandemic, as the two study periods coincided with different phases of the crisis. As highlighted by Celano et al. (2025) [18] in their comprehensive scoping review on vascular access during the pandemic, COVID-19 imposed unprecedented organizational stress on ICU workflows, forcing rapid changes in staffing ratios, compromising the strict maintenance of standard insertion and maintenance bundles, and leading to a global surge in healthcare-associated infections. Therefore, any implication of organizational equivalence between these two periods must be rejected. Group A (October–December 2020) was enrolled during the peak of the second pandemic wave in Italy, a period documented globally for a sharp rise in healthcare-associated infections—with national data reporting increases in CLABSI rates of up to 47%—due to extreme staff overload, increased patient acuity, and potential disruptions in standard catheter care bundles. This context likely explains the exceptionally high baseline infection rate observed in Group A (31.7%; 6.95 per 1000 catheter-days), suggesting that these results reflect systemic stressors rather than the failure of standard catheters alone. By framing this study as a before-and-after analysis under systemic stress, the transition to Group B in 2021 represents a period of comparative stabilization where the introduction of CSS technology served as a vital redundant defense mechanism during the recovery of healthcare protocols [18].

Group A (October–December 2020) was enrolled during the peak of the second pandemic wave in Italy, a period documented globally for a sharp rise in healthcare-associated infections—with national data reporting increases in CLABSI rates of up to 47%—due to extreme staff overload, increased patient acuity, and potential disruptions in standard catheter care bundles [19]. This context likely explains the exceptionally high baseline infection rate observed in Group A (31.7%; 6.95 per 1000 catheter-days), suggesting that these results reflect systemic stressors rather than the failure of standard catheters alone. Consequently, while our logistic regression model is statistically significant, it likely overestimates the isolated protective effect of the CSS coating (OR 0.15) as it could not fully adjust for these time-dependent risks. Crucially, because a multivariable regression or propensity score-adjusted approach was unfeasible due to missing granular database records (e.g., individual lumen numbers, emergency insertion ratios, or exact time-to-infection variables), the observed association may be partially driven by residual confounding. Future prospective trials utilizing multivariable modeling or randomized designs are necessary to confirm the exact causal effect size of CSS-coated CVCs. However, since baseline patient characteristics remained comparable between cohorts, the transition to Group B in 2021 represents a period of comparative stabilization where the introduction of CSS technology served as a vital redundant defense mechanism during the recovery of healthcare protocols [19].

Several studies have demonstrated that combining catheter impregnation with adherence to central line bundles synergistically decreases infection rates. Despite their higher initial cost, antimicrobial-impregnated catheters may be cost-effective in high-risk ICU populations by reducing CRBSI-related morbidity and length of stay. The use of antimicrobial-impregnated CVCs, particularly those coated with chlorhexidine–silver sulfadiazine, has consistently been associated with a significant reduction in CRBSI incidence compared with standard catheters [20].

A potential concern regarding CSS-coated catheters is the duration of their antimicrobial activity, which laboratory studies suggest may decline after the initial 48–72 h of use. However, clinical evidence suggests that the prevention of early bacterial colonization on the catheter surface is a critical factor in reducing the subsequent risk of mature biofilm formation and late-onset CRBSI [21,22]. In our cohort, despite a mean ICU stay of approximately 45 days, the CSS-coated catheters likely provided a crucial ‘protective window’ during the high-risk early post-insertion phase. Furthermore, the comparable ICU length of stay between Group A (45.7 days) and Group B (45.0 days) indicates that the observed reduction in CRBSI was not driven by earlier catheter removal in the CSS group, but rather by the efficacy of the antimicrobial coating in preventing the initial infectious trigger [21,22].

Current evidence indicates that antimicrobial CSS-coated CVCs reduce CRBSIs compared with uncoated catheters. However, no significant differences have been reported in terms of all-cause mortality or CRBSI-related mortality [23]. A recent meta-analysis estimated the mortality odds ratios of CLABSIs and CRBSIs, compared with uninfected patients, at 3.19 (95% CI: 2.44–4.16) and 2.47 (95% CI: 1.51–4.02), respectively [24].

Importantly, no significant differences in adverse effects—including thrombosis, bleeding, pain, pruritus, erythema, or local irritation—have been observed between impregnated and non-impregnated catheters [25]. Uncertainty remains regarding which type of antimicrobial coating is most effective. The two most widely used—chlorhexidine–silver sulfadiazine (CSS) and minocycline–rifampicin (MNR)—appear to have comparable efficacy [26].

Nevertheless, the emergence of antibiotic resistance among ESKAPE pathogens poses major challenges to both the prevention and treatment of CRBSIs. Multidrug-resistant organisms (MDROs) account for 20–67% of CRBSI cases, underscoring the urgent need for targeted infection control strategies. In CRBSIs, MDROs are independently associated with a higher risk: after adjustment for multiple confounders, the odds ratio for MDRO involvement was 4.63 (95% CI: 2.86–7.50; *p* < 0.001). The prevalence of MDRO infections was 3.9% in non-CRBSI patients compared with 62.3% in CRBSI patients (*p* < 0.001). These findings highlight the critical importance of specific management protocols to mitigate the impact of MDRO-associated CRBSIs.

In our ICU, infection prevention protocols for central venous catheters are based primarily on compliance with bundles and best practices [1]. These include maximal sterile barrier precautions (mask, cap, sterile gown and gloves, full-body drape), dedicated catheter-insertion carts or kits, strict hand hygiene, skin antisepsis with ≥2% chlorhexidine in alcohol, preferential use of the subclavian site over the femoral site, chlorhexidine dressings, daily chlorhexidine bathing, meticulous hub and cap decontamination, and the use of antiseptic- or antibiotic-impregnated catheters. In addition, patient-related risk factors such as immunosuppression, neutropenia, burns, malnutrition, obesity (BMI > 40), and prolonged hospitalization before catheter insertion must also be considered [27].

Despite these measures, the baseline historical incidence of **microbiologically confirmed CRBSI** in our ICU remains approximately 18.8%. The adoption of CSS-coated catheters was therefore motivated by evidence supporting their efficacy in further reducing infection rates [1,27]. Their safety profile is reassuring and comparable across different models, including with respect to the risk of anaphylaxis. Moreover, these catheters have been validated for compatibility with commonly used parenteral therapies over the past two decades [28,29,30].

This study has several limitations that should be acknowledged. First, its retrospective and observational design inherently introduces the risk of selection bias and limits the ability to establish causal relationships. While we attempted to mitigate this by comparing only those patients in both cohorts who shared the same high-risk clinical indications for CVC placement, the retrospective nature of the data meant that detailed information on several potential risk factors was not consistently available. Specifically, data on patient comorbidities (such as immunosuppression or renal failure), microbial colonization status, and standardized severity scores could not be fully incorporated into our analysis. Consequently, our logistic regression model primarily focused on catheter type and should be interpreted as an overall clinical trend (OR 0.15) rather than a comprehensive multivariate adjustment for all potential confounders. Furthermore, certain technical details—specifically the exact number of catheter lumens, the precise ratio of emergency versus elective insertions, the fixation techniques applied (sutures vs. sutureless systems), and the exact distribution of femoral versus non-femoral insertion sites—were missing or not systematically recorded in the database. As robustly demonstrated in the recent literature, such as the CONSIDER study by Borgonovo et al. (2025), these specific catheter-level factors act as major, independent predictors that significantly influence the risk of developing catheter-associated bloodstream infections [31]. Despite these gaps, the comparability of the two cohorts is supported by the absence of significant differences in baseline demographics, ICU admission diagnoses, and the consistency of institutional clinical protocols across both study periods. Nevertheless, because our retrospective analysis could not mathematically adjust for these crucial independent technical variables, their potential to confound or skew our rate estimates must be explicitly recognized as a significant limitation. Second, the comparison involved two different time periods (2020 and 2021), introducing a significant temporal bias. As discussed, the high baseline infection rate in 2020 likely reflects the unprecedented systemic stressors of the COVID-19 pandemic peak rather than the failure of standard catheters alone. Because we could not analytically adjust for this massive difference in organizational strain, the observed reduction in CRBSI rates must not be attributed solely to the technological superiority of the CSS coating, as the reduction is inextricably linked to the unmeasured alleviation of systemic crisis conditions in the ICU. Although no relevant changes occurred in ICU infection control policies or staff training, the transition from a crisis phase to a period of comparative stabilization represents a major confounding factor. Third, the study was conducted in only two ICUs within the same geographic area, and the observation was limited to two three-month intervals. This may limit the generalizability of the findings and prevented long-term assessment of outcomes, seasonal variations, or the development of potential antimicrobial resistance patterns. Finally, the lack of data on mortality, long-term morbidity, and formal cost-effectiveness, along with the absence of exact timing for CRBSI onset, prevented time-to-event analyses that could have provided deeper insights into the clinical and economic impact of CSS-coated catheters. Furthermore, utilizing ICU length of stay as a surrogate for catheter-days introduces the potential for non-differential misclassification of catheter exposure time. However, since this approximation was applied uniformly to both Group A and Group B using identical institutional criteria, any resulting misclassification would expectedly bias our rate estimates toward the null, thereby reinforcing the conservative nature of the observed protective effect. Future prospective, multicenter studies incorporating more granular variables as covariates are necessary to provide a more refined analysis.

## 5. Conclusions

Despite these limitations, the study provides clinically relevant evidence that, when targeted at high-risk patients according to international guidelines, CSS-coated CVCs are associated with a significant reduction in the incidence of microbiologically confirmed CRBSI and the number of catheter replacements in the ICU setting. Crucially, because of the study’s retrospective nature, the univariable statistical approach, and the drastically different phases of pandemic organizational strain characterizing the two cohorts, these results establish a historical association rather than definitive causation. Our findings suggest that in clinical environments where infection rates remain above institutional benchmarks despite bundle compliance, the selective use of antimicrobial-impregnated catheters represents an effective adjunctive strategy. These results support the need for future prospective, multicenter, randomized studies to further investigate long-term outcomes, potential resistance patterns, and the formal cost-effectiveness of this targeted approach.

## Figures and Tables

**Table 1:** Demographic and clinical characteristics of patients at ICU admission.

Variables	Total (N = 320)	Group A (N = 170)	Group B (N = 150)	*p*-Value
Age, Mean (SD)	54.9 (19.8)	54.3 (18.2)	55.4 (21.3)	0.67
Days in ICU, Mean (SD)	45.3 (11.6)	45.7 (12.1)	45.0 (11.1)	0.63
Catheter-days (Total)	14,519	7769	6750	-
Sex, Male (%)	175 (54.7%)	96 (56.7%)	79 (52.8%)	0.63
ICU admission pathology				0.43
Trauma (%)	76 (23.7%)	42 (25.0%)	34 (22.4%)	
Cerebral Hemorrhage (%)	67 (20.8%)	39 (22.5%)	29 (19.2%)	
Respiratory (%)	113 (35.5%)	54 (31.7%)	59 (39.2%)	
Others (%)	64 (20.0%)	35 (20.9%)	28 (19.2%)	

**Table 2:** Comparison of CRBSI incidence and catheter management between Group A and Group B.

Variables	Group A (N = 170)	Group B (N = 150)	*p*-Value
Confirmed CRBSI, n (%)	54 (31.7%)	10 (6.4%)	<0.0001
Infection rate per 1000 cat-days	6.95	1.48	<0.0001
Catheters removed, Median (IQR)	3 (2–4)	2 (1–2)	<0.0001

**Table 3:** Logistic regression analysis for the risk of developing CRBSI.

Comparison	Odds Ratio (95%CI)	*p*-Value
Group B vs. Group A	0.15 (0.06–0.32)	**<0.0001**

## Data Availability

All data are stored at the University of Campania “L. Vanvitelli”.

## Abbreviations

The following abbreviations are used in this manuscript:

CVC	Central venous catheters
CRBSI	Catheter-related bloodstream infection
IDSA	Infectious Diseases Society of America
CSS	chlorhexidine-silver sulfadiazine (CSS-coated antimicrobial CVC)
MNR	minocycline-rifampicin (CSS-coated antimicrobial CVC)
ICU	Intensive Care Unit
